# MHAU-Net: Skin Lesion Segmentation Based on Multi-Scale Hybrid Residual Attention Network

**DOI:** 10.3390/s22228701

**Published:** 2022-11-11

**Authors:** Yingjie Li, Chao Xu, Jubao Han, Ziheng An, Deyu Wang, Haichao Ma, Chuanxu Liu

**Affiliations:** 1 School of Integrated Circuits, Anhui University, Hefei 230601, China; 2 Anhui Engineering Laboratory of Agro-Ecological Big Data, Hefei 230601, China

**Keywords:** multi-scale resolution, hybrid residual attention, cross-validation, convolutional neural networks

## Abstract

Melanoma is a main factor that leads to skin cancer, and early diagnosis and treatment can significantly reduce the mortality of patients. Skin lesion boundary segmentation is a key to accurately localizing a lesion in dermoscopic images. However, the irregular shape and size of the lesions and the blurred boundary of the lesions pose significant challenges for researchers. In recent years, pixel-level semantic segmentation strategies based on convolutional neural networks have been widely used, but many methods still suffer from the inaccurate segmentation of fuzzy boundaries. In this paper, we proposed a multi-scale hybrid attentional convolutional neural network (MHAU-Net) for the precise localization and segmentation of skin lesions. MHAU-Net has four main components: multi-scale resolution input, hybrid residual attention (HRA), dilated convolution, and atrous spatial pyramid pooling. Multi-scale resolution inputs provide richer visual information, and HRA solves the problem of blurred boundaries and enhances the segmentation results. The Dice, mIoU, average specificity, and sensitivity on the ISIC2018 task 1 validation set were 93.69%, 90.02%, 92.7% and 93.9%, respectively. The segmentation metrics are significantly better than the latest DCSAU-Net, UNeXt, and U-Net, and excellent segmentation results are achieved on different datasets. We performed model robustness validations on the Kvasir-SEG dataset with an overall sensitivity and average specificity of 95.91% and 96.28%, respectively.

## 1. Introduction

Melanoma is a relatively aggressive form of skin malignancy that accounts for only about 1% of skin cancers but causes most deaths. There are currently more than 132,000 cases of melanoma skin cancer worldwide each year. The accuracy of diagnosis by patients and dermatologists by using visual inspection is only about 60%. In addition, the shortage of dermatologists per capita prompted the need for computer-aided methods in detecting skin cancer. The American Cancer Society’s 2022 homegrown statistics estimate that there will be approximately 99,780 new melanoma cases (about 57,180 cases in men and 42,600 cases in women) and an estimated 7650 deaths from melanoma (about 5080 men and 2570 women). In addition to this, there are other types of cancer. For example, colon cancer, lung cancer, stomach cancer, and so on are still the leading causes of human suffering and death.

**Figure 1:**
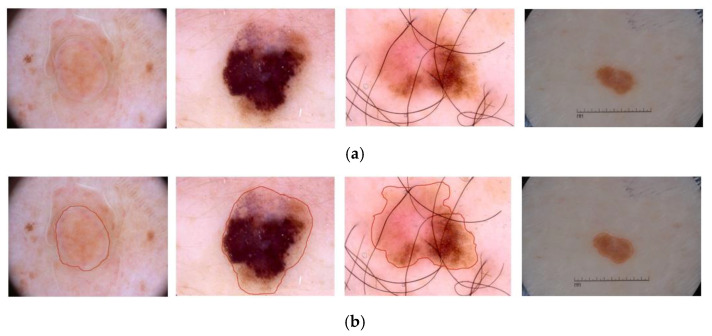
(**a**) Original dermoscopy images. (**b**) The red line area in the figure represents the lesion area of the corresponding image.

With the development of computer vision technology and artificial intelligence, image analyses have been widely used in various scene-parsing tasks. Medical image analyses play vital roles in computer-aided diagnosis and detection [1,2,3]. The amount of medical image data acquired is growing faster than the available human expert interpretation. Therefore, automated segmentation techniques are desired in helping physicians achieve accurate and timely imaging-based diagnoses [4,5]. However, due to insufficient original training samples of medical images or the lack of a clear demarcation line between some subtle lesion areas and normal tissues and organs (as shown in Figure 1). They are making the task of skin lesion segmentation more difficult.

In recent years, with in-depth research on deep learning theory, convolutional neural network-based [6,7,8,9] deep learning methods for image recognition and classification have shown excellent performance [10,11,12], including the recently popular BP neural network algorithm for image processing [13]. Moreover, with respect to multi-level dilated residual network [14] for processing skin lesions and MRIs, Long et al. [7] proposed an FCN architecture based on CNNs to solve the semantic level image segmentation problem by performing end-to-end pixel-level classification of the input raw images. Most medical images are large, so the feature vector obtained by training using raw images is large. It also has high requirements for computer performances, leading to substantial computational costs. Fischer et al. [15] proposed U-Net, which consists of mutually symmetric systolic and dilated paths. Among them, the systolic path is used to obtain context information and the dilated path is used for precise localizations. In the dilation path, feature vectors are fused with corresponding low-level features to add multi-scale information. Finally, the overlap tile strategy alleviates the computational resource issue. High IoU values of 0.9203 and 0.7756 were obtained on the PhC-U373 and DIC-HELa datasets, respectively. Later, Zhou et al. [16] proposed a new architecture U-Net++ that enables flexible feature fusion by redesigning multiple dense skip connections, reducing the semantic gap between feature representations and encoder sub-networks. Moreover, the multi-scale feature aggregation of U-Net++ can synthesize the segmentation results step by step, thus improving the accuracy and accelerating the convergence speed of the network.

## 2. Related Work

As the complexity of computer vision tasks and task demands increase, deeper [17] convolutional neural networks are required for feature extraction. As a result, the gradient disappearance problem sometimes occurs during feature propagation. Huang et al. [18] proposed Dense Net, which not only alleviates the gradient vanishing problem but also enhances the feature’s propagation and can dramatically reduce the number of parameters. Subsequently, Zhang et al. [19] proposed Res-Net, which utilizes skip connections (Identity mapping) to alleviate the vanishing gradient problem while increasing the network’s depth. The authors of [20] proposed PSP-Net using a pyramid pooling module to aggregate global contextual information from different regions to increase the target receptive field. Later, Ibtehaz et al. [21] proposed MultiResUNet to introduce contextual multi-scale information into the U-Net architecture via different residual modules, adding local detail information.

However, FCNs and CNN models face the same issue: a lack of long-term global correlation modeling capabilities. The main reason is that CNN extracts local information simply and cannot measure global relevance efficiently. A transformer [22] is an essential model in natural language processing, and was used initially to improve NMT (neural machine translation) models using attention mechanisms. The transformer network has a cleaner structure and is quicker in training and inferencing. The transformer focuses on extracting global information but weakens local information, so it also has some disadvantages in medical image segmentation tasks. How to properly highlight foreground information, weaken background information, and how to better jointly model local information and global correlation dependence become focuses of the study. The authors of [23] combined the transformer structure with the U-Net model, using the transformer’s powerful encoding ability and U-Net’s local localization ability to complete the segmentation of multiple abdominal organs and the heart. Extensive experiments demonstrate that TransU-Net outperforms the original U-Net architecture in various image classification tasks.

Based on existing approaches, in this paper, we propose a novel CNN for medical image segmentation. The training results on three different datasets outperformed the current state-of-the-art models in three main areas of work:

- Standard convolution is replaced by dilated convolution; original image information of varying resolution sizes is introduced into the encoder at all levels;
- Feature fusion at each level uses hybrid attention for detail enhancement of feature vectors in both channel and spatial dimensions;
- Slicing experiments are conducted to verify the contribution of HRA, dilation convolution, and cross-validation pieces relative to the MHAU-Net model.

**Image pre-processing** Since the lesion areas in the original dermoscopic images vary in shape, size, and pixel intensity, some lesion areas are hidden under human hair or shadows, which will inevitably affect segmentation results, thereby reducing the generalization ability of the model. Therefore, to minimize the impact of these factors on the model segmentation performance, we introduce an image preprocessing method.

**Figure 2:**
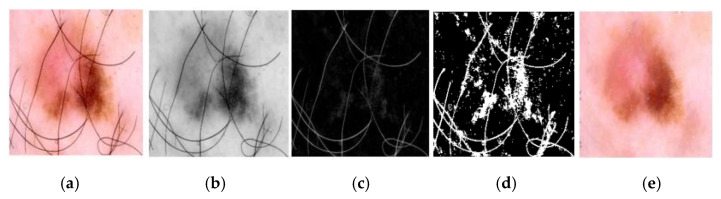
Sample of hair removal. (**a**) Original image (256 × 256). (**b**) Corresponding grayscale image. (**c**) Black hat image. (**d**) Thresholding image. (**e**) Inpainting.

We used a morphological manipulation approach to remove artifacts from the original dermoscopic images. First, the input RGB image is converted into a grayscale image. The morphological operation with black hat transform is used [24], followed by artifact removal using a thresholding operation (as shown in Figure 2 see legend information for details). We continuously adjusted the experimental parameters and selected a cross-shaped two-dimensional array of size 25 × 25 as the structural element, which has the middle row and column consisting of 1 and the remaining elements composed of 0. All images are resized to a shape of 256 × 256 using bilinear interpolation to achieve faster convolution operations and to solve the excessive memory consumption problem.

**K-Fold Cross-validation:** The medical image datasets that we acquired are limited, and the question of how to train models with high generalization performances on limited resources poses a new challenge to researchers. In this paper, we use a cross-validation strategy, which is also known as loop estimation. Cross-validation tests estimate the general validity of an algorithm on an independent data set, ensuring a balance between bias and variance. In the K-Fold cross-validation test, dataset D is randomly divided into k equal or nearly equal-sized mutually exclusive subsets D1, D2, …, Dk [25], which is then run k times, each time using one of the k blocks as the validation set and the rest as the training set. To evaluate the segmentation accuracy of the baseline U-Net and the proposed MSHMU-Net architecture, we perform a 5-fold cross-validation test on each of the different datasets.

## 3. Method

### 3.1. Attention Mechanism

In computer vision, attention mechanisms have been widely used in different task scenarios [26,27]. As an adaptive spatial region selection mechanism, spatial attention has been used in image classification [26] and image captioning [27], etc.

**Figure 3:**
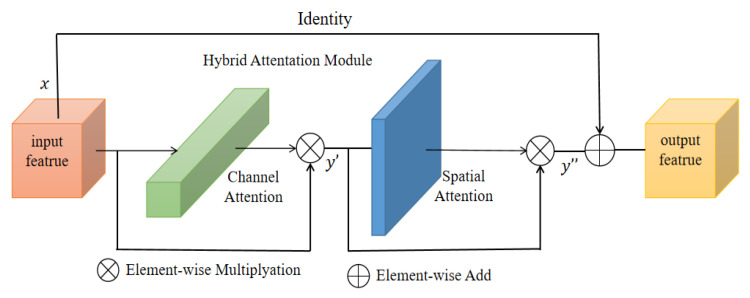
Hybrid residual attention.

To obtain a better segmentation output, we introduce a hybrid residual attention (HRA) module combined with identity mapping (as shown in Figure 3). First, the channel attention [28] module enhances the specific semantic responsiveness of channels by establishing associations between channels, thereby focusing on more meaningful parts. The second is the spatial attention module [29], which uses the association between any two point features to enhance the representation of their respective features mutually. Finally, the output results are added and fused to obtain the final features for pixel classification. The approach of these attention mechanisms is to achieve feature reinforcement by generating a context vector to assign the weights of the input sequence.

They take the input of feature vector $x\in R^{C*H*W}$ as an example. First, the channel attention mapping is used. Using both Max-Pool and Average-Pool algorithms, the transformation results are then obtained after several MLP layers and finally applied to two channels. The attention results of the channels are obtained using the sigmoid function (details of the operation are shown in Table 1). The output result is multiplied element-by-element with the original input feature vector (as shown in Equation (1)) to obtain the one-dimensional feature, $y^{\prime }\in R^{C*1*1}$. Next, the spatial attention mapping is used, and the two-dimensional spatial attention map $y^{\prime \prime }\in R{\;}^{1*H*W}$ is then obtained using the same method (as shown in Equation (2)). $M_{c}$ and $M_{s}$ represent the channel and spatial attention mapping operators, respectively.

(1) $$y^{\prime }=M_{c}\left(x\right)*x,$$

(2) $$y^{\prime \prime }=M_{s}\left(y^{\prime }\right)*y^{\prime }.$$

### 3.2. Residual Atrous Convolution

In general, the method for increasing the receptive field and reducing the amount of computation in deep neural networks is down-sampling. However, down-sampling sacrifices part of the spatial resolution and loses some information, which limits the effect of semantic segmentation. In contrast, atrous convolutions [30] enable effectively increasing target receptive field without increasing model parameters and without changing the size of the feature map. In addition, we introduce residual connectivity [10]. Residual connection not only reduces the complexity of model training to minimize overfitting but also prevents the gradient from vanishing. The RA convolutional network is proposed by combining the above two methods. In the RA module, we replace the standard convolution in the original CNN with dilated convolutions. On the one hand, the receptive field increases, and significant targets can be detected and segmented. On the other, the increased resolution compared with down-sampling can accurately locate the target. Combining residual connections can improve the mobility of information and prevent serious information loss. Significantly, RA can be integrated into other convolutional neural networks, which is a crucial reference for improving the propagation of feature vectors.

**Figure 4:**
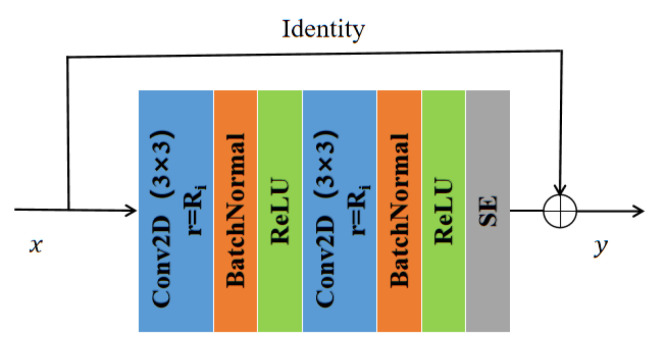
Residual atrous convolution.

For the input feature vector, two 3 × 3 dilated convolutions (as shown in Figure 4) followed by normalization were used to prevent the occurrence of gradient explosions. Then, a rectified linear unit was used for activations to alleviate the overfitting problem. Finally, an identity mapping and squeeze excitation unit [28] was introduced to add and fuse the output with the original feature vector.

### 3.3. MHAU-Net Architecture

**Coding Phase:** The encoding stage uses RA blocks with different dilation rates for feature information extraction (as shown in Table 1). The RA block uses a 3 × 3 convolution with stride 2 instead of pooling during down-sampling. To avoid overfitting and underutilizing resources, after each layer of convolution operation, the feature maps of each layer are normalized using a batch normalization layer and then activated using the leaky ReLU activation function (as shown in Figure 4). Then, the feature vectors are input into HRA, and the dependencies between channels are established. Using the dependency relationship between feature channels, the feature representation of specific semantics can be improved to generate channel attention maps. The spatial attention module encodes a vast range of contextual information into local features, thus enhancing their expressive power. The spatial relations among the elements generate a spatial attention graph. HRA has powerful feature representation capabilities that can be integrated into other CNN architectures. However, frequently using channels and spatial attention mechanisms increases spatial and time complexities. The high resolution low-level features and the smaller field of perception of individual pixels enable the use of more fine-grained feature information to capture more small targets. The validation shows that increasing too many attention mechanisms does not bring about significant improvements but instead increases the training burden. Therefore, we choose to use attention mapping after three more low-level features of RA, R_3_A, and R_4_A.

**Figure 5:**
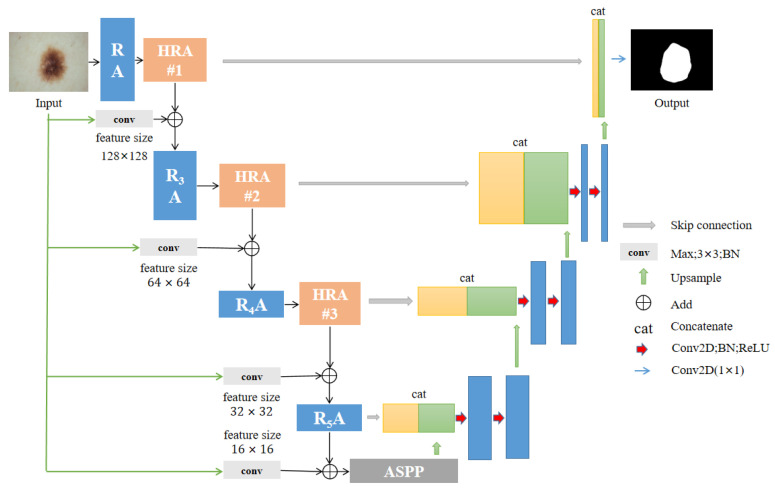
Our proposed MHAU-Net network architecture. RA represents the residual dilated module (as shown in Figure 4). HRA means hybrid residual attention, and it is used three times in total.

Meanwhile, we perform four 3 × 3 pooling convolution operations with different steps on the original image. Images with varying resolution sizes are input to each encoder level in a multi-scale manner and are encoded with multi-scale contextual information (as shown in Figure 5). Given an original image with a size of 256 × 256, after four sampling operations, images of sizes 128 × 128, 64 × 64, 32 × 32, and 16 × 16 are obtained, respectively, and then added to the feature vector of the corresponding coding level.

**Figure 6:**
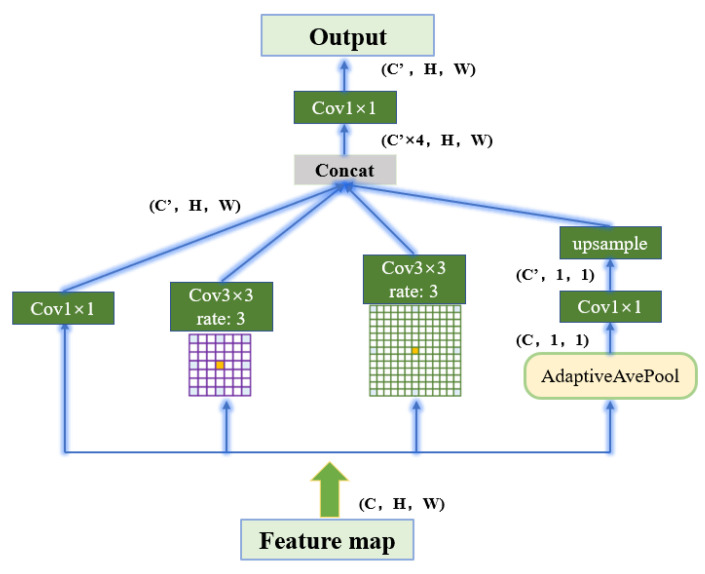
Atrous spatial pyramid pooling.

**Transition Phase:** ASPP is composed of a 1 × 1 convolution (shown on the far left in Figure 6), a pooling pyramid (two 3 × 3 convolution blocks in the middle), and an adaptive pooling layer (far right). The dilation factor of each layer of the pooled pyramid can be customized, and different scales of perceptual fields can be obtained by extra padding and dilation. The advantage of using AdaptiveAvgPool2d layers is that there is no need to assign a convolution kernel and step size, as only the final output size needs to be specified. The purpose is to compress the feature maps of each channel to 1 × 1, respectively, to extract the features of each channel and thus obtain the global features.

**Decoding Phase:** The decoding path adopts the exact opposite operation of the encoding path. First, the output features of ASPP are up-sampled using bilinear interpolation with a step size of 2. The output features corresponding to the encoder level are extracted and spliced with up-sampling features. Second, feature reduction is performed using convolutions of size 3 × 3, followed by batch normalization and then followed by activation operations. Finally, a 1 × 1 convolution and a sigmoid activation function are applied to output mask features.

## 4. Experiments and Results

### 4.1. Datasets

**Figure 7:**
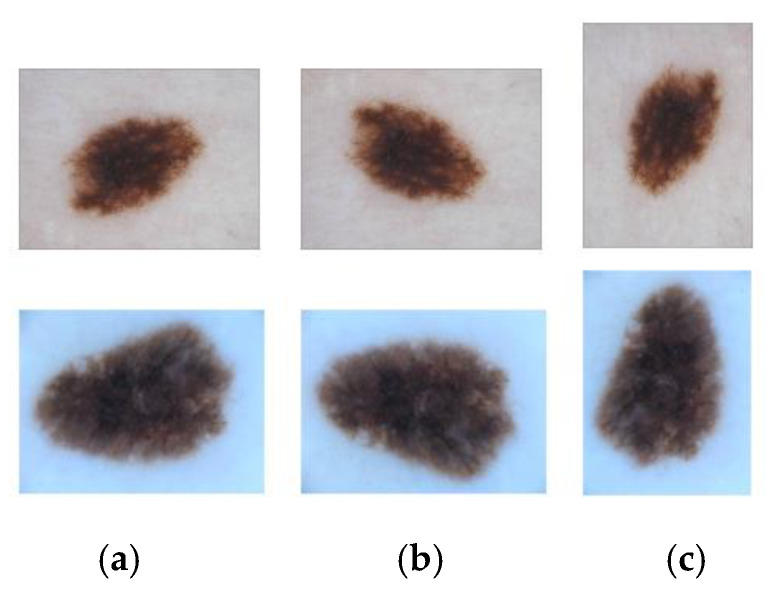
(**a**–**c**) are the results of the original, vertical flip, and transposition of the two images, respectively.

To evaluate MHAU-Net, we conducted experiments on three public medical image datasets. In this paper, data augmentation techniques, including vertical flip and transpose (as shown in Figure 7), were used in advance for all datasets participating in the experiments (details of the data set are shown in Table 2). However, we do not establish the validation dataset. Since we use a cross-validation strategy, some data are randomly divided as the validation set in each training round. Cross-validation enables an increase in the randomness of the validation dataset and the training parameters are adjusted in time, thus effectively improving the generalization performance of the model.

### 4.2. Evaluation Metrics

In this paper, we use the standard metrics commonly used for semantic segmentation to demonstrate that MHAU-Net has a more accurate segmentation output than other popular models. The Dice Similarity Coefficient (DSC) (as shown in Equation (3)) is used to evaluate the similarity between the segmentation output and the actual labels, and the value ranges from [0, 1]; the larger the value, the higher the similarity between the two sets. The Intersection over Union (IoU) is the ratio of the intersection of the true and predicted values of a prediction category to the union (as shown in Equation (4)). The sensitivity (SEN), defined as Equation (5), indicates the proportion of correctly segmented lesion pixels, and high sensitivity (close to 1.0) shows a good segmentation effect. Specificity (SPE) (as shown in Equation (6)) indicates the proportion of non-lesioned skin pixels that are not correctly segmented. The high specificity suggests the ability of the method to segment non-lesioned pixels:

(3) $$DSC=\frac{2*TP}{\left(2*TP+FP+FN\right)},$$

(4) $$IoU=\frac{TP}{TP+FN+FP},$$

(5) $$Sensitivity=\frac{TP}{TP+FN},$$

(6) $$Specificity=\frac{TN}{FP+TN},$$

(7) $$IoU=\frac{DSC}{2-DSC},$$

where the relationship between *DSC* and *IoU* can be expressed as Equation (7). The *IoU* of each prediction category is found and the output mIoU is obtained by taking the average value. *TP* is the True Positive. *FP* is the False Positive. *FN* is the False Negative. *TN* is the True Negative.

### 4.3. Experimental Configuration

All experimental programs are implemented in the PyTorch 1.11.0 framework and run on a single-core NVIDIA GeForce RTX 3090 with a 24 GB dedicated GPU. A stochastic gradient descent optimization strategy is used, with an initial learning rate of 10^−3^ and a learning rate reduction of 1/5 for every 15 epochs. The batch size and the number of epochs are set to 16 and 150, respectively.

### 4.4. Results

**Figure 8:**
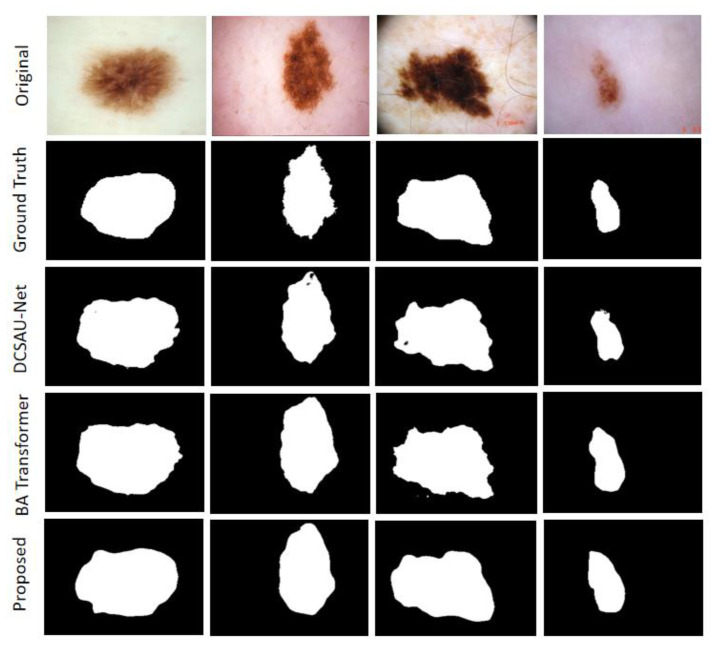
ISIC 2018 Segmentation output sample.

This section presents the segmentation results of the MAHU-Net method exhibited in different datasets. Quantitatively, in the ISIC 2018 Task1 challenge dataset, our proposes has better segmentation performances compared to the original U-Net and the latest DCSAU-Net [31] architectures, with 7.67% and 4.91% improvements in mIoU, respectively, and DSC compared to DCSAU-Net with a 3.41% improvement compared to DCSAU-Net. It was 93.9%, 92.7%, 94.69%, and 87.92% in overall sensitivity, specificity, DSC, and mIoU, respectively; DSC, mIoU, and SPE were superior to DCSAU-Net by 3.41%, 4.91%, and 3.11%, respectively (as shown in Table 3). The case of the final segmentation of our proposed method on the ISIC 2018 validation set is provided in Figure 8.

**Figure 9:**
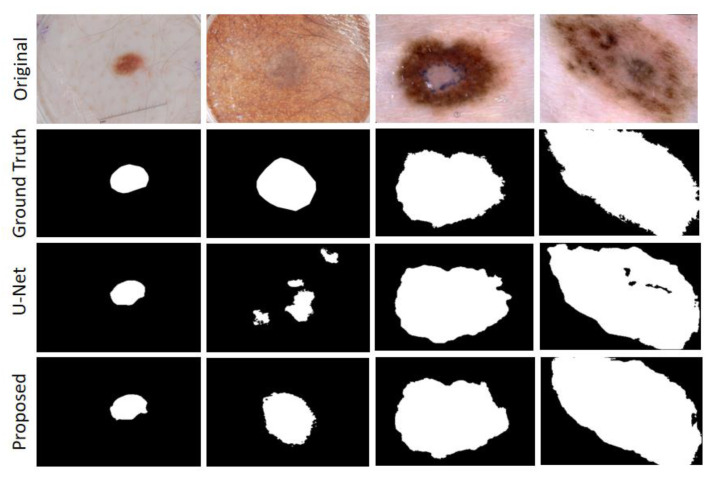
ISIC 2017 Segmentation output sample: the third row shows the resulting graph of U-Net.

On ISIC-2017 Task1, we merged 150 images data from the validation set into the training set and then used data augmentation techniques. Finally, the results are derived using the cross-validation strategy. The overall sensitivity and specificity outperformed the original U-Net by 2.47% and 3.01%, respectively (as shown in Table 4). The case of the final segmentation of our proposed method on the ISIC 2017 task validation set is provided in Figure 9.

**Figure 10:**
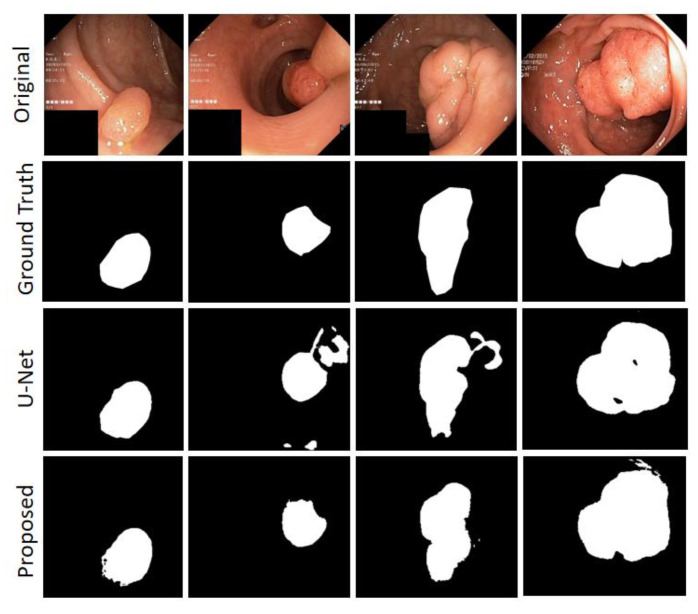
Kvasir-SEG Segmentation output sample.

On the Kvasir-SEG dataset, we compared it with the currently popular Double U-Net, Pra-Net, and U-Net. The results show that our method is highly competitive. DSC is 1.34%, 1.92%, and 9.92% higher than Double U-Net, Pra-Net, and U-Net, respectively; it only has a 0.12% difference from the TransFuse-S architecture. However, we show the large advantage with a high mIoU of 0.9025. SEN is 1.65% and 6.72% higher than U-Net and Double U-Net, respectively (as shown in Table 5). An experimental comparison between U-Net and our method is given in Figure 10, and our proposal shows a more robust output in tiny tissue regions.

The comparative experimental data in Table 3, Table 4 and Table 5 are from the original article cited, and the code is publicly available on GitHub.

### 4.5. Slice Experimental

In this paper, we use a slice experimental approach to evaluate the contribution of HRA and other vital components to semantic segmentation. The experiments use the original U-Net as the segmentation baseline to verify the gains from repeated HRA, dilated residual convolution, and cross-validation experiments. The same hyperparameter settings are used for all experiments. ISIC 2018 Task1 is used as an example to illustrate the effect of these network components. Starting with the baseline model U-Net, experiments were progressively performed with HRA #1, HRA #2, and HRA #3. For mIoU, applying HRA #1 improves segmentation performances by 1.38%, using both HRA #1 and HRA #2 improves it by 3.71%, and using HRA #1, HRA #2, and HRA #3 together improves it by 4.92% (as shown in Table 6). The segmentation performance is raised by 5.84% by adding dilation convolution. Lastly, the model generalization performance is boosted using cross-validation methods.

The end experimental results indicate that MHAU-Net demonstrates good performance compared to the original U-Net and the recently popular Double U-Net, DSCAU-Net, and MFS-Net in different metrics. From the training results in Table 6, it can be seen that SPE has a decreasing trend. This error may be related to the increase in false positives (i.e., classifying some non-lesioned pixels as lesioned pixels). However, in terms of experimental results, the training result of MHAU-Net is about 9% higher than that of U-Net. It provides a reference for the improvement of subsequent network models.

## 5. Discussion and Future Work

The MHAU-Net architecture proposed in this paper achieved satisfactory results from ISIC-2018 Task1, ISIC-2017, and Kvasir-SEG dataset. From the information shown in Figure 8, Figure 9 and Figure 10, it could be concluded that the segmentation maps generated by the MHAU-Net outperformed the other architectures in capturing the boundary information, demonstrating that the segmentation masks generated in the MHAU-Net showed more precise information in the target area than the existing models. The full convolutional network has more room for improvements in capturing skin lesion locations and edge details.

In this paper, we combine the binary cross-entropy loss function and the dice loss function to train the proposed model. With the same loss function, the proposed model achieves higher dice coefficient values than the other models. Based on the empirical evaluation, the dice coefficient loss function is chosen to achieve better segmentation results. In addition, the effects of batch size, optimizer, and loss function selection on the results are observed.

We speculate that the performance of the model can be further improved by increasing the size of the dataset, applying more enhancement techniques, and applying some post-processing steps. The application of MHAU-Net should not be limited to biomedical image segmentation; it can also be extended to natural image segmentation and other pixel-level classification tasks that require further detailed validation. The proposed method could be feasible for future medical imaging analyses and clinical exam routines.

Future improvements will include the following:

1. We attempt to develop an enhanced version of MHAU-Net for video analyses in the medical field.
2. Future studies will integrate the hybrid residual attention module proposed in this paper in other skin lesion segmentation models to verify its enhancement of the model’s results.

## 6. Conclusions

Inspired by U-Net and the attention mechanism, we propose MHAU-Net to address the need for the automated detection of lesion areas in dermoscopy and its related medical fields. MHAU-Net consists of four parts: multi-scale resolution input, hybrid residual attention (HRA), dilated convolution, and atrous spatial pyramid pooling. HRA fully utilizes the benefits of the attention mechanism to achieve feature enhancements and conducts slicing experiments to verify the contribution of network components to the overall network architecture. Use a 5-fold cross-validation strategy during training to improve the generalization performance of the model. We validated MHAU-Net on three datasets with better performance than the BA-Transformer and U-Net. To achieve the goal of model generalizability, the proposed architecture in this paper should be further investigated for improvements to obtain better segmentation results.

## Figures and Tables

**Table 1:** MHAU-Net architecture details.

Block	Layer (Filter Size)	Block	Layer (Filter Size)
RA	Conv2D (3 × 3), rate = 1	R_4_A Block3	Conv2D (3 × 3), rate = 4
	Batch Normal, ReLU		Batch Normal, ReLU
	Conv2D (3 × 3), rate = 1		Conv2D (3 × 3), rate = 4
	Batch Normal, ReLU		Batch Normal, Re LU
	SE Module		SE Module
R_3_A Block2	Conv2D (3 × 3), rate = 3	R_5_A Block4	Conv2D (3 × 3), rate = 5
	Batch Normal, ReLU		Batch Normal, ReLU
	Conv2D (3 × 3), rate = 3		Conv2D (3 × 3), rate = 5
	Batch Normal, ReLU		Batch Normal, ReLU
	SE Module		SE Module
conv	Max pooling (2 × 2)	ASPP	Conv2D (1 × 1) and (3 × 3) AdaptiveAvgPool2d
	Conv2D (3 × 3)		
	Batch Normal		Upsample
Channel Attention	MaxPool	Spatial Attention	MaxPool
	AveragePool		AveragePool
	**MLP, Sigmoid**		**Concatenate, Conv2D**

**Table 2:** Details of the medical segmentation datasets used in our experiments.

Datasets	Original Resolution	Input Resolution	Train	Test
ISIC-2018	Variable	256 × 256	7782	1000
ISIC-2017 Task1	Variable	256 × 256	6450	600
Kvasir-SEG	Variable	256 × 256	2700	100

**Table 3:** Comparison of results of different network architectures on ISIC 2018 Task1 dataset.

Network	DSC	mIoU	SEN	SPE
**U-Net** [15]	-	0.8025	0.8659	0.9128
**U-NeXt** [32]	0.8970	-	-	-
**BA Transformer** [33]	0.9120	0.8430	-	-
**DCSAU-Net** [30]	0.9128	0.8301	-	0.8959
**Proposed**	**0.9469**	**0.8892**	**0.939**	**0.9270**

“-” indicates that the relevant public data are not available.

**Table 4:** Comparison of results of different network architectures on the ISIC 2017 Task1 dataset.

Network	DSC	mIoU	SEN	SPE
**U-Net** [15]	0.774	0.8389	0.9081	0.9009
**MFS-Net** [34]	**0.974**	**0.987**	0.9257	-
**Proposed**	0.9523	0.9615	**0.9328**	**0.931**

“-” indicates that the relevant public data are not available.

**Table 5:** Comparison of results of different network architectures on the Kvasir-SEG dataset.

	DSC	mIoU	SEN	SPE
**U-Net** [15]	0.8180	0.7640	0.8919	-
**PraNet** [35]	0.8980	0.8490	-	-
**Double U-Net** [36]	0.9038	0.8816	0.9426	0.9571
**TransFuse-S** [37]	**0.9180**	0.8680	-	-
**Proposed**	0.9172	**0.9025**	**0.9591**	**0.9628**

“-” indicates that the relevant public data are not available.

**Table 6:** Evaluation of different component settings on the ISIC 2018 Task1 dataset.

Network Setup	Dice	mIoU	SEN	SPE
**U-Net**		0.8025	0.8359	0.9328
**+HRA#1**	0.8261	0.8163	0.8526	0.9289
**+HRA#1and#2**	0.8593	0.8396	0.8598	0.9302
**+HRA All**	0.9038	0.8517	0.8668	0.9253
**+Dilate Conv**	0.9256	0.8609	0.9085	**0.9456**
**+5-Fold**	**0.9469**	**0.8892**	**0.9391**	0.9270
